# Bridging the gap: access to health care and control of type 2 diabetes mellitus, hypertension and bronchial asthma among Egyptian women

**DOI:** 10.1186/s12889-025-21638-2

**Published:** 2025-02-05

**Authors:** Eman Ibrahim Elmeshmeshy, Basma Abdelaziz, Saeed Soliman

**Affiliations:** Faculty of Medicine, Cairo University, Cairo, Egypt

**Keywords:** Access to health care, Diabetes mellitus, Hypertension and bronchial asthma, And Control status

## Abstract

**Background:**

Non-communicable diseases burden is rising globally; women are disproportionately affected by both non-communicable diseases burden and lack of access to health care. Research on the relationship between access to health care and non-communicable diseases control are limited. We aimed to assess the relationship between women’s access to health care and three non-communicable diseases control.

**Methods:**

A cross-sectional study was conducted on 420 women diagnosed with diabetes mellitus, hypertension and/or Asthma. Access to health care was assessed through pretested validated Arabic version of access 31 questionnaire. The control status of diabetes mellitus, hypertension, and asthma was assessed according to the American Diabetes Association HbA1c targets, World Health Organization Guidelines, and the Global Initiative for Asthma control assessment tool, respectively. The associations were assessed using univariate and multivariate logistic regression analysis.

**Results:**

(83%) of the participants had poor access to health care, of those, (39.0%) achieved non-communicable diseases control, while (16.9%) participants had good access to health care, of them, (83.1%) had their non-communicable diseases controlled. This difference in non-communicable diseases control proportions across access to care groups was statistically significant (*P* < 0.001). Women with good access to health care had higher odds (Adjusted Odds Ratio 6.33, 95% confidence interval: 3.24–12.55) of having their non-communicable diseases controlled compared with those with poor access.

**Conclusion:**

Improving access to health care is essential for achieving better non-communicable diseases control.

**Supplementary Information:**

The online version contains supplementary material available at 10.1186/s12889-025-21638-2.

## Introduction

Achieving Universal health coverage (UHC) is one of the sustainable development goals set by the nations of the world to be achieved by 2030. UHC ensures that people and communities have access to the required health service when and where they need it [1]. 

Non-communicable diseases (NCDs) account for 41 million deaths annually and 74% of all deaths globally. These diseases include diabetes, cancer, chronic respiratory conditions, cardiovascular disease, and respiratory disorders. Asthma is as important as diabetes and hypertension as a major (NCD) and most asthma-related deaths occur in low- and lower-middle-income countries. Asthma diagnosis, treatment, and monitoring are all being improved by the World Health Organization (WHO) in an effort to lower the worldwide burden of NCDs and achieve (UHC). NCDs are additionally the main cause of illness and mortality for both men and women. Almost everywhere worldwide, they have also been a significant factor in the deaths and disabilities of women [2]. 

In Egypt, 84% of all deaths in 2016 were due to NCDs [3]. Patients with NCDs have a desperate need for health care, and access to adequate healthcare services is an important aspect for them [4]. 

Access to healthcare is not merely service availability; it includes five dimensions of access, i.e. (a) availability, (b) accessibility, (c) affordability, (d) accommodation and (e) acceptability [5]. 

Disparities in access to and utilization of healthcare services globally are one of the most serious challenges facing the healthcare system, and as a result, there have been a lot of studies interested in figuring out the causes of this disparity [6]. NCDs suffers from this disparity in access and the resulting detrimental effects on health. It represents a major public health priority in NCDs control [7]. 

Access to affordable preventive, early detection, diagnosis, treatment, and care of NCDs is particularly difficult for girls and women who are living with these conditions, especially in poor nations. Significant obstacles include ingrained poverty, gender inequality, the stigma attached to NCDs, women’s household duties, and the expense of getting care. Access to resources and the ability to make independent decisions about their use are crucial for women’s ability to afford NCD healthcare.

In many poor nations, women’s sociocultural standing results in less health knowledge and access to resources [8]. 

50% of the Egyptian population is women [9]. Inequity in health outcomes, access to healthcare and the financial burden of healthcare are well-documented difficulties facing Egypt’s health system [3]. In this regard, we conducted this research, The research hypothesis is that access to health care may affect NCDs control, especially for women, and our research aims to find out the relationship between women access to health care and its association with NCDs control.

## Methods study design and setting

This cross-sectional study was conducted from December 2022 to December 2023 in family medicine, internal medicine, endocrinology, and chest outpatient clinics at Cairo University Hospitals.

### Participants

#### Inclusion and exclusion criteria

Women aged 18–60 years diagnosed with one or more of the following NCDs: Diabetes mellitus (DM), Hypertension (HTN) and/or Asthma for more than one year were eligible for enrollment, while women who were pregnant, intellectually disabled and those with known psychiatric disorders were excluded.

### Sampling size and sampling technique

Based on a previous study assessing the prevalence of access to health care for NCDs among Egyptian women 54.4% [10]. Open Epi^®^ software was used to calculate the sample size of the cross-sectional study (frequency in the population). Assuming, 54.4% anticipated frequency, 80% power, 0.05 level of significance, and 95% confidence level, the sample size was calculated to be = 382 participants, considering drop-outs and incomplete response rate of 10%,; therefore the final sample size has been raised to 420 participants.

A convenient sampling technique was applied to recruit women attending the mentioned outpatient clinics and meeting the inclusion criteria until the required sample size of 420 participants was reached.

### Data collection

An informed written consent was obtained from each participant. The research team collected data through face-to-face interviews using a structured questionnaire. The questionnaire assessed socio-demographic characteristics (age, marital status, employment, and education), access to health care and NCDs diagnosis and achieved targets. Access to healthcare was assessed using the Arabic version of the Access 31 questionnaires. This is a tool that was developed, validated and pre-tested in Omani population. It focuses on 6 aspects of access namely: (1)Geographical access: (travel time to the primary health care (PHC), transportation options available, transportation costs), (2) Information accessibility (the availability of data on test findings, health issues, and the promptness of responses to inquiries both during and after work hours), (3) Organizational access (appointment availability, clinic wait times, appointment wait times, and phone accessibility), (4)Affordability (cost of medication and consultation), (5) Cultural acceptability (Language, health information, privacy, and respect) and (6)Availability of services and medicines (Discussing prescription drugs, the standard of care, blood test availability, and scheduling visits with a general practitioner, specialists, and other medical personnel) [11]. 

For each item, a response of “No problem” received a score of 0 (the patient has no issue, problem or obstacle to access to healthcare in this domain) while “problem” response received a score of 1 (the patient has an issue or obstacle to access to healthcare in this domain). Hence, the range of domains scores (by calculating minimum and maximum subdomain scores) was 0–3 for geographical access, 0–5 for information access, 0–4 for organizational access, 0–2 for affordability, 0–6 for cultural acceptability, and 0–5 for the availability of services and medications. Each domain’s score was the total of the individual question scores.

A respondent’s overall access score was obtained by adding the scores of these 6 dimensions, giving a range of 0 to 25. The median (50th percentile) score of all the variables was calculated with a cutoff value of 12. Accordingly; it was dichotomized into poor access when the score was > 12 and good access if it was ≤ 12. Based on 50th percentiles split, the 50th -percentile split, also known as the median split involves dividing the data into two groups at the median value. It is a common practice in statistical analysis to simplify the interpretation of results [12]. 

The control status of DM, HTN and asthma was assessed according to American Diabetes Association HbA1c targets [13], (WHO) Guidelines [14] and the Global Initiative for Asthma (GINA) [15], respectively. Uncontrolled diabetics were those with HbA1c > 8.0%.^13^ For HTN, uncontrolled hypertensive patients were those with blood pressure (BP) ≥ 140/90.^14^ Patients should achieve these targets for at least six months for DM and one month for both HTN and asthma.

As participants might have one of those 3 diseases or more than one, we used the following operational definition for NCDs control status: For those having one disease, they were considered controlled or uncontrolled according to their affecting disease status e.g. If the patient had only DM that was controlled, his control status was controlled. Those having more than one, they were considered uncontrolled if any one of the affecting diseases was uncontrolled. While those having both of their affecting diseases controlled were considered controlled e.g. If the patient had controlled DM and uncontrolled HTN, his control status was considered uncontrolled. Also, the patient having controlled DM and controlled HTN, his control status was considered controlled.

### Statistical analysis

Descriptive statistics (frequency and percentage for nominal and categorical variables and median and interquartile (IQR) or mean and standard deviation (SD) for continuous data based on a normality check) were used to summarize participants’ characteristics, access to health care score and status, and NCDs control status.

Bivariate association using the chi-square test and (Fisher exact test for expected cell count less than 5). Kruskal Wallis test was used to compare access scores across NCDs control categories. Binary logistic regression models were used to examine the association between access to healthcare and NCDs control, adjusting for potential confounding variables (age, employment status, family size, and educational level). These variables were selected for inclusion in the adjusted model based on literature review and less than 0.2 significance in the univariate associations with NCDs control. Odds ratios (OR) and 95% confidence intervals (CI) were reported. P value less than 0.05 was considered statistically significant [16]. 

## Results

In summary a diagnosis of any of the three diseases HTN, DM and /or Asthma were included in the analysis. They had a median age (IQR) of 50 (19:60) years. Most of them (*n* = 294, 70.0%) lived in rural areas, were married (*n* = 286, 68.1%), and unemployed (*n* = 294, 70.0%). More than half of them had more than 3 offsprings (*n* = 227, 54.0%), and had their NCDs not controlled (*n* = 225, 53.6%) as shown in Table 1. The NCDs control status was significantly associated with employment status, number of offsprings and educational level: being more controlled in women with 3 children or less, who had a job or business and had a higher educational level.

**Table 1:** Basic demographic characteristics of the study population by NCDs control status (*n* = 420)

	NCDs control status			
	Not controlled (*n* = 225, 53.6%)	Controlled (*n* = 195, 46.4%)	Total (*n* = 420)	*P* value^*#*^
**Age**				
≤ 50 years	109 (48.4%)	110 (56.4%)	219 (52.1%)	0.103
>50 years	116 (51.6%)	85 (43.6%)	201 (47.9%)	
**Residence**				
Rural	68 (30.2%)	54 (27.7%)	122 (29.0%)	0.659
Suburban	27 (12.0%)	20 (10.3%)	47 (11.2%)	
Urban	130 (57.8%)	121 (62.1%)	251 (59.8%)	
**Employment**				
Has no job	172 (76.4%)	122 (62.6%)	294 (70.0%)	0.002*
Has job	53 (23.6%)	73 (37.4%)	126 (30.0%)	
**Marital status**				
Married	152 (67.6%)	134 (68.7%)	286 (68.1%)	0.575
Widow/separated	56 (24.9%)	42 (21.5%)	98 (23.3%)	
Never married	17 (7.6%)	19 (9.7%)	36 (8.6%)	
**Offsprings**				
≤3 offsprings	109 (48.4%)	118 (60.5%)	227 (54.0%)	0.013*
>3 offsprings	116 (51.6%)	77 (39.5%)	193 (46.0%)	
**Educational level**				
No formal education	28 (12.4%)	11 (5.6%)	39 (9.3%)	< 0.001*
Basic (Primary/preparatory)	127 (56.4%)	79 (40.5%)	206 (49.0%)	
Secondary education and above	70 (31.1%)	105 (53.8%)	175 (41.7%)	

NCDs: Non-communicable disease

**Fig. 1:**
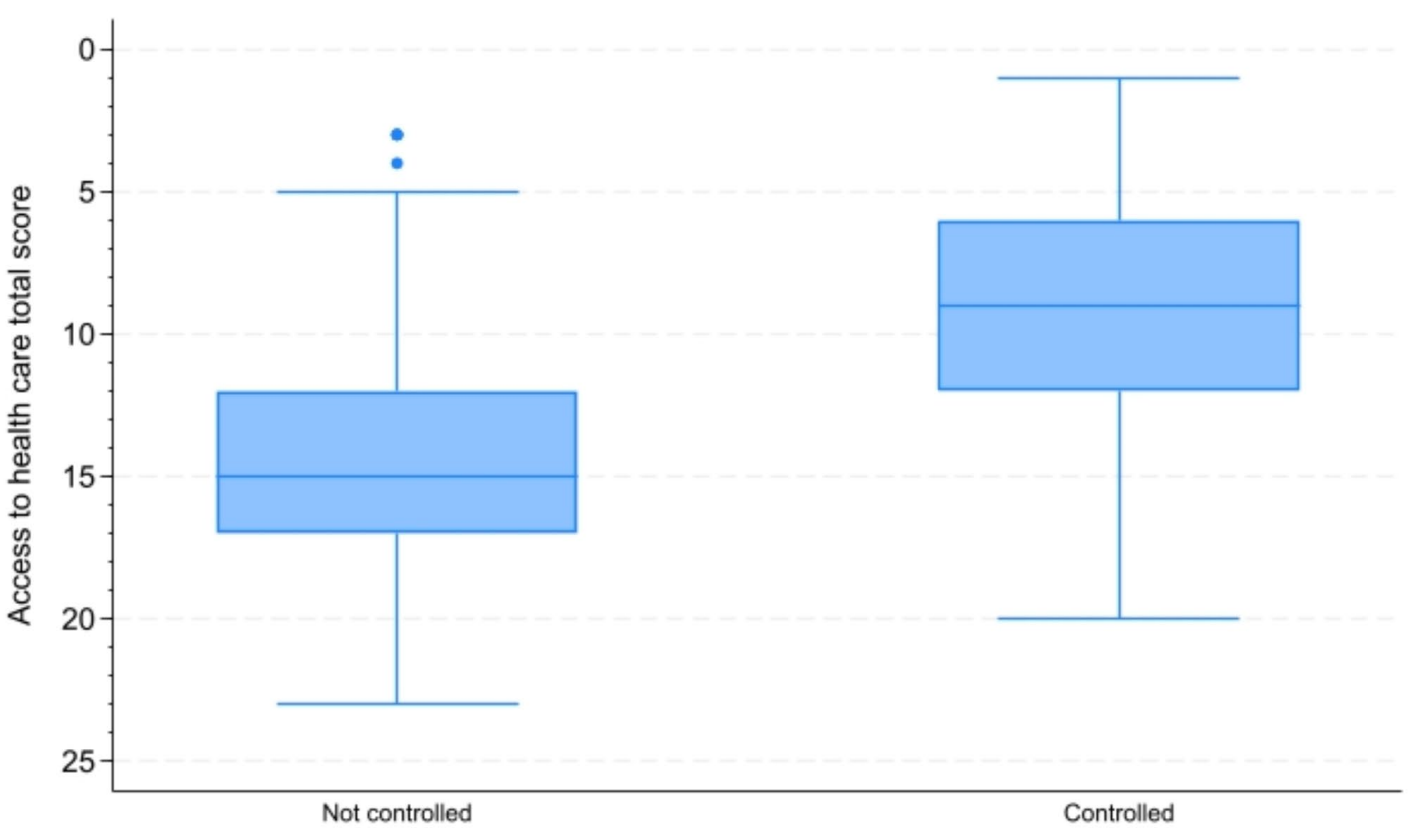
Comparison of access to health care scores between controlled and not controlled groups

Figure 1 shows the distribution of access to care total scores across controlled and uncontrolled groups, Consequently, the total access score median (IQR) was higher in the non-controlled group (15; IQR12: 17) compared with the controlled group (9, IQR 6: 12), with *P* < 0.001.

Regarding the six domains of access to health care, the affordability domain had the highest proportion of reporting “no issues” by 53.6% of participants (*n* = 225), followed by cultural acceptability (*n* = 92, 21.9%) and geographical access (*n* = 79, 18.8%). Furthermore, there were statistically significant differences between the NCDs-controlled and non-controlled groups in all domains, except for organizational access. The controlled group reported fewer issues in most domains, especially in affordability, cultural acceptability, and availability of services and medicines, as shown in Table 2.

**Table 2:** Scores of access to health care domains and their relation to NCDs control status

NCD control status				
	Not controlled (*n* = 225)	Controlled (*n* = 195)	Total (*n* = 420)	*P* Value
**Geographical access**				
0	39 (17.3%)	40 (20.5%)	79 (18.8%)	**< 0.001**
1	106 (47.1%)	126 (64.6%)	232 (55.2%)	
2	53 (23.6%)	21 (10.8%)	74 (17.6%)	
3	27 (12.0%)	8 (4.1%)	35 (8.3%)	
**Access to information**				
0	7 (3.1%)	12 (6.2%)	19 (4.5%)	**< 0.001**
1	7 (3.1%)	22 (11.3%)	29 (6.9%)	
2	10 (4.4%)	39 (20.0%)	49 (11.7%)	
3	53 (23.6%)	82 (42.1%)	135 (32.1%)	
4	38 (16.9%)	17 (8.7%)	55 (13.1%)	
5	110 (48.9%)	23 (11.8%)	133 (31.7%)	
**Organizational access**				
0	11 (4.9%)	21 (10.8%)	32 (7.6%)	**0.015**
1	99 (44.0%)	87 (44.6%)	186 (44.3%)	
2	32 (14.2%)	38 (19.5%)	70 (16.7%)	
3	61 (27.1%)	32 (16.4%)	93 (22.1%)	
4	17 (7.6%)	16 (8.2%)	33 (7.9%)	
5	5 (2.2%)	1 (0.5%)	6 (1.4%)	
**Affordability**				
0	77 (34.2%)	148 (75.9%)	225 (53.6%)	**< 0.001**
1	43 (19.1%)	21 (10.8%)	64 (15.2%)	
2	105 (46.7%)	26 (13.3%)	131 (31.2%)	
**Cultural acceptability**				
0	15 (6.7%)	77 (39.5%)	92 (21.9%)	**< 0.001**
1	30 (13.3%)	48 (24.6%)	78 (18.6%)	
2	54 (24.0%)	30 (15.4%)	84 (20.0%)	
3	63 (28.0%)	25 (12.8%)	88 (21.0%)	
4	43 (19.1%)	14 (7.2%)	57 (13.6%)	
5	16 (7.1%)	0 (0.0%)	16 (3.8%)	
6	4 (1.8%)	1 (0.5%)	5 (1.2%)	
**Availability of services and medicines**				
0	0 (0.0%)	20 (10.3%)	20 (4.8%)	**< 0.001**
1	26 (11.6%)	49 (25.1%)	75 (17.9%)	
2	40 (17.8%)	47 (24.1%)	87 (20.7%)	
3	49 (21.8%)	41 (21.0%)	90 (21.4%)	
4	61 (27.1%)	28 (14.4%)	89 (21.2%)	
5	36 (16.0%)	8 (4.1%)	44 (10.5%)	
6	13 (5.8%)	2 (1.0%)	15 (3.6%)	

NCDs: Non-communicable disease

Table 3. shows the association between access to health care and NCDs control status. The most frequent NCDs was DM (*n* = 244, 66.0%) followed by HTN (*n* = 240, 57.0%) and asthma (*n* = 72, 17.1%). The vast majority of the 420 participants had poor access to care (*n* = 349, 83%), of those, 136 (39.0%) achieved NCDs control, while 71 (16.9%) participants had good access to health care, of them, 59 (83.1%) had their NCDs controlled. This difference in NCDs control proportions across access to care groups was statistically significant (*P* < 0.001).

**Table 3:** DM, HTN and asthma control and their association with access to health care among study participants, *n* = 420

	Access to health care			
	Poor access (*n* = 349)	Good access (*n* = 71)	Total	*P* value
**DM**				
Not present	158 (45.3%)	38 (53.5%)	196 (46.7%)	< 0.001
Controlled	55 (15.8%)	26 (36.6%)	81 (19.3%)	
Not controlled	136 (39.0%)	7 (9.9%)	143 (34.0%)	
**HTN**				
Not present	148 (42.4%)	32 (45.1%)	180 (42.9%)	< 0.001
Controlled	100 (28.7%)	34 (47.9%)	134 (31.9%)	
Not controlled	101 (28.9%)	5 (7.0%)	106 (25.2%)	
**Asthma**				
Not present	293 (84.0%)	55 (77.5%)	348 (82.9%)	0.559
Controlled	24 (6.9%)	8 (11.3%)	32 (7.6%)	
Partially controlled	20 (5.7%)	5 (7.0%)	25 (6.0%)	
Not controlled	12 (3.4%)	3 (4.2%)	15 (3.6%)	
**NCDs control status**				
Not controlled	213 (61.0%)	12 (16.9%)	225 (53.6%)	< 0.001
Controlled	136 (39.0%)	59 (83.1%)	195 (46.4%)	

DM: Diabetes mellitus HTN: Hypertension NCDs: Non-communicable diseases

Univariate and multivariate logistic regression analyses of the association of access to care and NCDs control are shown in Table 4. In the adjusted model, women who have good access to health care have higher odds (AOR 6.37, CI:3.24–12.55) of NCDs control compared with those have poor access. In addition, women who have secondary education and higher have higher odds (AOR 2.64, CI:1.17–5.96) of NCDs control compared to no education.

**Table 4:** Univariate and multivariate logistic regression analysis of predictors of NCDs control (n *=* 420)

	Univariate			Multivariate			
	OR	95% CI	*p*-value	OR	95% CI	*p*-value	
**Access**							
Poor access	1			1			
Good access	7.7	[3.99,14.85]	**> 0.001**	6.33	[3.24,12.55]	**> 0.001**	
**Age**							
≤50 years	1			1			
>50 years	0.73	[0.49, 1.07]	0.1035	0.90	[0.58, 1.39]	0.635	
**Offsprings**							
≤3 offsprings	1			1			
>3 offsprings	0.61	[0.42, 0.90]	**0.014**	0.88	[0.57, 1.36]	0.561	
**Educational level**							
No education	1			1			
Basic (Primary/preparatory)	1.58	[0.75, 3.36]	0.231	1.70	[0.77, 3.71]	0.187	
Secondary education and above	3.82	[1.79, 8.17]	**0.0006**	2.64	[1.17, 5.96]	0.0198	
**Employment**							
Has no job	1			1			
Has job	1.94	[1.27, 2.96]	**0.0021**	1.52	[0.95, 2.43]	0.079	

NCDs: Non-communicable diseases OR: Odds ratio CI: confidence interval AOR: Adjusted Odds Ratio

*Multivariate logistic regression model adjusted for children number, education, and employment status

## Discussion

In our study, the vast majority of women (83%) had poor access to health care. This agrees with **Chiang et al.** study that reported that Women’s utilization of health care was still low in the underdeveloped south of Egyptian society [17]. Also, the Central Agency for Public Mobilization and Statistics (CAMPAS) estimates that 46.9% of females were identified to be health insurance members or recipients in 2017 in contrast to 54.6% of male beneficiaries [18]. 

The most noticeable feature of the findings was that this poor access was unlikely to be due to affordability, cultural and geographical obstacles as the affordability domain had the highest proportion of reporting “no issues” followed by cultural acceptability and geographical access. This can be explained by that most of study participants who were from urban areas (low geographic accessibility barriers) and highly educated (no cultural barriers). Similar results were reported by **Lopes Ibanez-Gonzalez & Norris** cross-sectional study. They found that around 85.8% of the respondents had at least one type of healthcare service available within a 2 km radius of their homes. Of this group, 18% felt that these services were unaffordable [19]. 

In contrast to these findings, a study on women in Ghana’s rural and urban areas has found that income and distance have a significant impact on how often women in the Ahafo-Ano South district use health services (p value = 0.007) [20]. Another one conducted in the United States found that uninsured chronically ill patients were more likely than those insured to have not visited health professionals (22.6% vs. 6.2%) and to have a standard site for care [21]. Healthcare provider preference, income, waiting time, and distance to the health center were found to be significant (p-value < 0.05) in a cross-sectional study that looked at the various factors affecting healthcare utilization for NCDs among the rural and urban households of Indian district**s** [22]. In this study, the barriers have been faced were related to access to information, medication and services availability and organization access respectively.

Additionally, the control group reported fewer issues in most domains, especially affordability, cultural acceptability, and availability of services and medicines. This goes in accordance with the elements set to integrate NCDs management in primary care with adjustments to governance, service organization, and financing. Creating standards, educating healthcare professionals, and guaranteeing access to vital medications and tests are important to improving NCDs outcomes [23]. 

In our study, there was a significant association in bivariate analysis between NCDs control and access to health care. In the adjusted model, women who have good access to health care have higher odds (AOR 6.33, CI:3.22–12.44) of NCDs control compared with those who have poor access. This is consistent with a cross-sectional study of diabetes patients in Oman, in which following confounder adjustment, the odds of having non-controlled glycated hemoglobin were 2.6 times greater than those in the controlled group as shown by the access composite score [11]. Chronic illness affecting women is a barrier to getting the recommended care according to the available data on women’s experiences of chronic care within health care systems [24]. 

In our study, we found that NCDs control is associated with employment status. According to the results of the 2019 Behavioral Risk Factor Surveillance System among adults in the United States, which supports our findings, the prevalence of all adverse health outcomes were highest for those who were unable to work, followed by those who were unemployed for a long time and then for a brief period; the prevalence were lowest for those who were employed or self-employed. Over 40% reported having, high blood pressure and high cholesterol level [25]. Furthermore, a study of how NCDs affect employment status in South Africa showed that NCDs posed a great threat to women’s work status [26]. However, medication adherence was considerably higher among unemployed individuals (*p* = 0.008) in cross-sectional research of Omani patients with NCDs [27]. 

In this study, NCDs control is positively associated with education level for secondary and above education. This is similar to **Menon et al.** The study results in which a statistically significant correlation was observed between the occurrence of DM among women and low educational status [28]. Additionally, in an investigation into the relationship between educational attainment and mortality as well as NCDs, adjusted analyses revealed that people with at least some college experience were less likely to develop chronic illnesses; for the most part, the odds of these common disease conditions were inversely correlated with educational attainment, except for obesity [29]. Well-educated patients have better information, find alternatives to their treatment and are more aware of health promotion and prevention. They also have the probability of finding a good and well-paid job. Therefore, education leads to higher income and consequently to fewer barriers to access.

### Limitations

This study is one of the countable studies that assesses the association between access to health care, and NCDs control. However, our study has some limitations worth acknowledging, including, the cross-sectional design which doesn’t allow the establishment of causal relationships between control of NCDs and access to health care, but rather only associations. Also, it does not differentiate between the types of the services being private or public (governmental).

### Recommendations

The present findings would contribute to the policy formulation in reducing health inequity issues in terms of increasing women’s access to care by enabling women to get equal rights to education and jobs.

One of the suggested strategies on the national level is expanding universal health insurance coverage to overcome barriers in several access domains and to improve access to health services among vulnerable groups.

## Conclusions

Our study highlights that potentially poor NCDs control is linked with poor access to health care. The cause for this poor access was neither the unaffordability of the services nor the geographical inaccessibility. Both good access to health care and high education level are predictors for NCDs control. The major obstacles to access to health care in patients with NCDs were information, medication and service availability. Stockholders in Egypt have to implement integrated approaches for NCDs management in primary care and improving consultation and communications between the healthcare providers and the patients with NCDs.

## Electronic supplementary material

Below is the link to the electronic supplementary material.

Supplementary Material 1

## Data Availability

The datasets used and/or analysed during the current study are available from the corresponding author on reasonable request.

## Abbreviations

UHC: Universal health coverage
NCD: Non-communicable diseases
WHO: World Health Organization
DM: Diabetes mellitus
HTN: Hypertension
PHC: Primary health care
BP: Blood pressure
GINA: Global Initiative for Asthma
IQR: Inter quartile range
SD: Standard deviation
CI: Confidence intervals
OR: Odds ratios
AOR: Adjusted odds ratio
CAMPAS: Central Agency for Public Mobilization and Statistics
