# Supplementary material for: Bridging the gap: access to health care and control of type 2 diabetes mellitus, hypertension and bronchial asthma among Egyptian women
Source: BMC Public Health. 2025 Feb 5;25:466. doi: 10.1186/s12889-025-21638-2 (PMC11800612; doi:10.1186/s12889-025-21638-2)
Supplement: Supplementary file 1 — Supplementary Material 1 [file 12889_2025_21638_MOESM1_ESM.docx]

| **Access to health care barriers in women with NCDs.**  **Table 1. Geographical access and Access to information in studied women with**  **NCDs *(n*=420)** | | | | | | | |
| --- | --- | --- | --- | --- | --- | --- | --- |
|  |  |  |  |  |  |  | **Number (%)** |
| **Geographical access** | | | |  |  |  |  |
| **Commutation to health center** | | | |  |  |  |  |
|  | Car |  |  |  |  |  | 57 (13.6%) |
|  | Walking |  |  |  |  |  | 21 (5%) |
|  | Public transport | |  |  |  |  | 338 (80.5%) |
|  | Other |  |  |  |  |  | 4 (0.95%) |
| **Time to reach health center** | | | |  |  |  |  |
|  | <30 min |  |  |  |  |  | 94 (22.4%) |
|  | 30-60 min |  |  |  |  |  | 211 (50.2%) |
|  | 1-3 hours |  |  |  |  |  | 112 (26.7%) |
|  | >3 hours |  |  |  |  |  | 3 (0.7%) |
| **Visit cancel because there is no transport** | | | | |  |  |  |
|  | Yes |  |  |  |  |  | 48 (11.4%%) |
|  | No |  |  |  |  |  | 372 (88.6%) |
| **Visit cancel because there is no money for transport** | | | | | |  |  |
|  | Yes |  |  |  |  |  | 102 (24.3) |
|  | No |  |  |  |  |  | 318 (75.7%) |
| **Access to information** | | | |  |  |  |  |
| **Doctor or health center call to inform lab results** | | | | | |  |  |
|  | Yes |  |  |  |  |  | 79 (18.8%) |
|  | No |  |  |  |  |  | 341 (81.2%) |
| **Advice about how to stay healthy** | | | |  |  |  |  |
|  | Yes |  |  |  |  |  | 243 (57.9%) |
|  | No |  |  |  |  |  | 177 (42.1%) |
| \| **Table 1. Geographical access and Access to information barriers in studied women with NCDs *(n*=420) continue.** \| \| --- \| | | | | | | | |
| **Number (%)** | | | | | | | |
| **Advice about health concern** | | | |  |  |  |  |
|  | Yes |  |  |  |  |  | 241 (57.4%) |
|  | No |  |  |  |  |  | 179 (42.6%) |
| **Health center answers call during office hours** | | | | | |  |  |
|  | Yes |  |  |  |  |  | 85 (20.2%) |
|  | No |  |  |  |  |  | 335 (79.8%) |
| **Health center answers call after office hours** | | | | |  |  |  |
|  | Yes |  |  |  |  |  | 35 (8.3%) |
|  | No |  |  |  |  |  | 385 (91.7%) |

| **Table 2. Cultural acceptability and affordability in studied women with NCDs (*n*=420).** | | | | | | | | | | |  |
| --- | --- | --- | --- | --- | --- | --- | --- | --- | --- | --- | --- |
|  |  |  |  |  |  |  |  | **Number (%)** | | |  |
| **Cultural acceptability** | | | |  |  |  |  |  |  |  |  |
| **Understand doctor language** | | | |  |  |  |  |  |  |  |  |
|  | Yes |  |  |  |  |  |  | 389 (92.6%) |  |  |  |
|  | No |  |  |  |  |  |  | 31 (7.4%) |  |  |  |
| **Past difficulty to understand doctor language** | | | | |  |  |  |  |  |  |  |
|  | Yes |  |  |  |  |  |  | 88 (20.9%) |  |  |  |
|  | No |  |  |  |  |  |  | 332 (79.1%) |  |  |  |
| **Received written information about illness** | | | | |  |  |  |  |  |  |  |
|  | Yes |  |  |  |  |  |  | 187 (44.5%) |  |  |  |
|  | No |  |  |  |  |  |  | 233 (55.5%) |  |  |  |
| **Private room while discussing health condition** | | | | |  |  |  |  |  |  |  |
|  | Yes |  |  |  |  |  |  | 158 (37.6%) |  |  |  |
|  | No |  |  |  |  |  |  | 262 (62.4%) |  |  |  |
| **Treated with disrespect during visit** | | | |  |  |  |  |  |  |  |  |
|  | Yes |  |  |  |  |  |  | 57 (13.6%) |  |  |  |
|  | No |  |  |  |  |  |  | 345 (82.1%) |  |  |  |
|  | Don’t know | |  |  |  |  |  | 18 (4.23%) |  |  |  |
| **Health concerns answers in satisfied manner** | | | | |  |  |  |  |  |  |  |
|  | Yes |  |  |  |  |  |  | 262 (62.4%) |  |  |  |
|  | No |  |  |  |  |  |  | 158 (37.6%) |  |  |  |
| **Affordability** | | |  |  |  |  |  |  |  |  |  |
| **Table 2. Cultural acceptability and affordability in studied women with NCDs (*n*=420) continue.** | | | | | | | | | | | |
| **Number%** | | | | | | | | | | | |
| **Postponed visit due to unaffordability** | | | | |  |  |  |  |  |  |  |
|  | Yes |  |  |  |  |  |  | 160 (38.1%) |  |  |  |
|  | No |  |  |  |  |  |  | 260 (61.9%) |  |  |  |
| **Stopping medication due to unaffordability** | | | | |  |  |  |  |  |  |  |
|  | Yes |  |  |  |  |  |  | 166 (39.5%) |  |  |  |
|  | No |  |  |  |  |  |  | 254 (60.5%) |  |  |  |

| **Table 3. Organizational barriers in studied women with NCDs (*n*=420)** | | | | | | | | | |
| --- | --- | --- | --- | --- | --- | --- | --- | --- | --- |
|  |  |  |  |  |  |  |  | **Number (%)** | |
| **Duration taken to talk to doctor** | | | |  |  |  |  |  |  |
|  | 0-10 min |  |  |  |  |  |  | 75(17.9%) | |
|  | 10-30 min |  |  |  |  |  |  | 41 (9.8%) |  |
|  | 30-60 min |  |  |  |  |  |  | 9 (2.1%) |  |
|  | >hour |  |  |  |  |  |  | 5 (1.2%) |  |
|  | Can’t talk |  |  |  |  |  |  | 290 (69.1%) | |
| **Evening and weekend appointments** | | | | |  |  |  |  |  |
|  | Yes |  |  |  |  |  |  | 71 (16.9%) | |
|  | No |  |  |  |  |  |  | 349 (83.1%) | |
| **Appointment needs to health center** | | | |  |  |  |  |  |  |
|  | Yes |  |  |  |  |  |  | 157 (37.4%) | |
|  | No |  |  |  |  |  |  | 263 (62.6%) | |
| **If yes Waiting duration of an appointment** | | | |  |  |  |  |  |  |
|  | 1-2 days |  |  |  |  |  |  | 19 (4.5%) |  |
|  | 3-7 days |  |  |  |  |  |  | 12 (2.9%) |  |
|  | 7-30 days |  |  |  |  |  |  | 53 (12.6%) | |
|  | >month |  |  |  |  |  |  | 73 (17.4%) | |
| **Waiting duration to see a doctor** | | | |  |  |  |  |  |  |
|  | less than 15 min | |  |  |  |  |  | 118(28.1%) | |
|  | 15-30 min |  |  |  |  |  |  | 155 (36.9%) | |
|  | 30-45 min |  |  |  |  |  |  | 83 (19.8%) | |
|  | 45-60 min |  |  |  |  |  |  | 35 (8.3%) |  |
|  | >hour |  |  |  |  |  |  | 29 (6.9%) |  |
| **Consultant referrals need** | | |  |  |  |  |  |  |  |
|  | Yes |  |  |  |  |  |  | 118 (28.1%) | |
|  | No |  |  |  |  |  |  | 302 (71.9%) | |
| **If yes Waiting duration to see a consultant** | | | | |  |  |  |  |  |
|  | 1-2 days |  |  |  |  |  |  | 63 (15 %) |  |
|  | 3-7 days |  |  |  |  |  |  | 45 (10.7%) | |
| \| **Table 3. Organizational barriers in studied women with NCDs (*n*=420) continue.** \| \| --- \| \| **Number (%)** \| | | | | | | | | | |
|  | 7-30 days |  |  |  |  |  |  | 7 (1.7%) |  |
|  | >month |  |  |  |  |  |  | 2 (0.5%) |  |
| **Ability to get health service in case of injury** | | | | |  |  |  |  |  |
|  | Yes |  |  |  |  |  |  | 40 (9.5%) |  |
|  | No |  |  |  |  |  |  | 97 (23.1%) | |
|  | Not applicable | |  |  |  |  |  | 283 (67.4%) | |
|  |  |  |  |  |  |  |  |  |  |

| **Table 4.**  **Access to health care in study sample of women with**  **NCDs (*n*=420) (Availability of services and medicines)** | | | | | | | | | |  |  |
| --- | --- | --- | --- | --- | --- | --- | --- | --- | --- | --- | --- |
|  |  |  |  |  |  |  |  | **Number (%)** | | |  |
| **Received information about medication** | | | | |  |  |  |  |  | |  |
|  | Yes |  |  |  |  |  |  | 293 (69.7%) |  | |  |
|  | No |  |  |  |  |  |  | 127 (30.2%) |  | |  |
| **Specific health center patient consult** | | | | |  |  |  |  |  | |  |
|  | Yes |  |  |  |  |  |  | 253 (60.2%) |  | |  |
|  | No |  |  |  |  |  |  | 167 (39.8%) |  | |  |
| **Patient didn’t receive prescribed medicine** | | | | |  |  |  |  |  | |  |
|  | Yes |  |  |  |  |  |  | 64 (15.2%) |  | |  |
|  | No |  |  |  |  |  |  | 225 (53.6%) |  | |  |
|  | Physician didn’t ask | |  |  |  |  |  | 131 (31.2%) |  | |  |
| **Availability of labs** | | |  |  |  |  |  |  |  | |  |
|  | Yes |  |  |  |  |  |  | 194 (46.2%) |  | |  |
|  | No |  |  |  |  |  |  | 71 (16.9%) |  | |  |
|  | Physician didn’t ask | |  |  |  |  |  | 155 (36.9%) |  | |  |
| **Availability of dietitian or physiotherapist** | | | | |  |  |  |  |  | |  |
|  | Yes |  |  |  |  |  |  | 60 (14.3%) |  | |  |
|  | No |  |  |  |  |  |  | 61 (14.5%) |  | |  |
|  | Physician didn’t ask | |  |  |  |  |  | 299 (71.2%) |  | |  |
| **No doctor available** | | |  |  |  |  |  |  |  | |  |
|  | Yes |  |  |  |  |  |  | 352 (84.05) |  | |  |
|  | No |  |  |  |  |  |  | 65(15.5%) |  | |  |
|  |  |  |  |  |  |  |  |  |  | |  |
